# We Made Better Dentists Then Than Now

**Published:** 1903-12

**Authors:** D. R. Stubblefield

**Affiliations:** Nashville, Tenn.


### "WE MADE BETTER DENTISTS THEN THAN NOW."[1]

BY D. R. STUBBLEFIELD, M.D., D.D.S., NASHVILLE, TENN.

THIS startling quotation is from one of the best men in American Dentistry. For more than twenty years he has been admired as a man, a teacher, and a dentist, one whose life traditions gave warrant of excellent ability, whose aspirations considered the highest attainment only as worthy, and whose associations argued

[1] Read before the Academy of Stomatology, Philadelphia, March, 1903.

conclusively that his profession might look upon him with confidence and pride.

The occasion of the utterance was a meeting of teachers of dentistry, and the special subject of discussion was the practical adjustment of the work of the schools to the new order of things which now confronts us as a profession. I say *confronts us as a profession,* for it seems all but axiomatic to say that the profession must be vitally concerned in everything connected with the institutions which educate its rank and file. The old men must jealously guard and wisely counsel them, and the young men must loyally defend and enthusiastically encourage them, for no profession can hope to rise above the fountains from which its current flows.

On the above occasion it had been claimed that the addition of another college year gave a much needed opportunity to add certain and sundry important phases of modern scientific knowledge to the college curriculum. The enthusiast who proposed had been followed by others, until the roseate dawn of a newer and better era in dentistry was flung upon the imaginary canvas of our professional East and the kindling enthusiasm of the moment was beginning to spread like any other conflagration. Then it was that this certain man spoke among other things the sentiment which has been considered worthy of your interest to-night. He is no sentimentalist. He said what he did from no desire to detract from the dignity and worth of what is done to-day, from the stand-point of intrinsic scientific value, but he gave the sober judgment of a thoughful mind. For more than twenty-five years he has satisfactorily filled the demands of an intelligent, progressive teacher, and from this eminence, this coign of vantage, having surveyed the whole field, he uttered the conviction that *better dentists were made in the past than to-day.*

Is it true? Is it only partly true? Or is it without foundation in fact? Do you young men believe that those who preceded you came upon the field of professional life better equipped, better able to discharge its duties, than you who may rightly claim to be the exponents of a later, more progressive *régime?* In the light of this day of boasted scientific inquiry and investigation it is a bold, if not a rash man who can utter such a sentiment. But, again, I declare that the man who said it is worthy of your confidence; in truth, that man holds your confidence to-day, and is entitled to that distinction from every single step between sentiment and sense.

I come, therefore, demanding the right of trial by jury, demanding a hearing for the reason of his faith. This question is too vital to the profession to be left unanswered. Although it is unthinkable that any living profession can ever become fixed and unalterable, crystallized in one state from which there can be no change short of constitutional disintegration, at the same time, right now the supreme legislative body of our institutions of instruction has demanded a change which must shake the methods of our colleges to their foundations. It behooves us, therefore, to reason together, to listen to each other in the utterance of honest convictions, to reserve judgment until all of the evidence is in, and wisely decide our future course. In the language of another, "Whilst yet the storm thunders in the distance let us sound the waters beneath us, ascertain our true position, and determine our future course." From this Academy of Stomatology, from this great city, one of the acknowledged centres of professional confidence, must come some declaration to assist in making a wise, even a monumental professional conclusion.

If you should say this is no affair of ours, that it is a question that the colleges alone must answer, you reckon without your host. The college is nothing but the concrete exponent of what the profession at large demands. Whether a man wishes it or not, when he enters a profession right then he assumes its obligations and cannot shut his eyes nor close his ears to its claims upon him. Therefore I reassert that, whether you wish it or not, whether you were conscious or not in assuming it, you have constituted yourself the sponsor of your professional schools by assuming the obligations of your chosen calling. The college is and has been just what you demand it shall be. It is no more perfect, nor infallible, nor worthy of praise or censure, than you yourselves, for it is bone of your bone and flesh of your flesh. Therefore, the condition, the demand, that confronts it confronts you, and you must help to carry the praise or odium of its wise or unwise solution. Shall more time mean, also, more material in the course? Shall it be interpreted as an opportunity to more thoroughly digest what is already crowding the curriculum? Is this the time to take stock of our methods, to scrutinize the claims of each part of the composite course, to readjust instruction with reference to relative time in the course, fitting it more accurately to the maturity of mind of the students engaged? One man has spoken, let others speak out. In the multitude of council we may get wisdom.

But before others may have the floor, what can be said about this proposition that "We made better dentists then than now"? If he is correct, a clear understanding of his reasons for belief may be of much value in settling the problem of the four-year law. For the purpose of this discussion let me assume the responsibility of his utterance and offer myself awhile, at least, as the victim upon the altar of investigation. I say this in sound mind and disposing memory, with a full consciousness of the enormity of the offence and with a just apprehension of the dire fate that may await me. The sergeant-at-arms has been duly instructed to lock and bar the doors from the outside, and no man of you can escape hearing this suicidal confession, even if it causes the marrow of your bones to freeze and the hair of your flesh to stand up with chilling horror. I am determined to do it, even if I, reportorily, scatter my brains and spatter my heart's hot blood upon your innocent heads. I, who am about to die, salute you!

In all seriousness, hear his cause. There is some foundation in fact to the claim that better dentists were made in other years than right now. From the stand-point of an interested observer for almost a quarter of a century, I am ready to believe that the modern introduction of so much or so many varied branches of science has tended to obscure the clearness of sight and definiteness of grasp upon the great fundamental idea of all dental instruction. It may be asked, Do you mean to antagonize progress and modern scientific development? Do you attempt to deny that the wealth of scientific contribution to the course of instruction has enriched the minds of those upon whom it has been lavished? If not, how can you hope to maintain such a conclusion that is all but iconoclastic in its effect upon that awe-inspiring pile of knowledge, otherwise known as the up-to-date dental college curriculum? I cannot deny its richness, its wealth of scientific attainment, and for that reason I do not make my vicarious substitution unconditional. But I do claim that such profundity of knowledge, such wealth of science, do not necessarily give the coming dentist a commensurate degree of excellence in rendering practical the golden idea of greatest fundamental importance and make him of necessity the ideal exponent of its greatest beneficence to mankind.

To my mind, the cart is before the horse. If such extensive acquisition of scientific knowledge could be made to exert its enriching power before the student has set himself to acquire that perfect technic which alone can make him the ideal practitioner, it would be better. Then the proper relation between scientific learning and practical performance would be better established. Then the student would, indeed, be more able to see broadly and decide maturely the application of means to ends and the scientific reason for them.

If it be true that the introduction of even valuable material may obscure the vision or the practical simplicity of the end aimed at, then I hold that the instruction of other years which was not interfered with by what may be called an embarrassment of riches was able to produce a stronger, clearer practitioner than the graduate of to-day can possibly claim to be at the outset of his career. A practitioner of any calling is essentially a *doer.* It is, *therefore,* unthinkable that any exponent of a practical utility can be unable to do, to make, to accomplish. This idea could be illustrated in every field of human enterprise, but the bare statement of it here is sufficiently axiomatic for our purpose. Just so long as the college course held as its highest, if not its only ideal the attainment of power to do, it was naturally productive of a better development in that direction. At that time its appropriation of really valuable knowledge from cognate sciences, was dominated by the ruling idea of perfect practicality. Such outside influences, if they did not obviously better it, were not allowed to attain to anything like a dangerous extent from the stand-point of that practical end in view. If I may use a homely figure, just so long as these scientific outsiders were used as condiments or flavoring extracts, they contributed to the general good effect of the whole, but when they became, or were dangerously near becoming, a preponderating component, they spoiled the whole pudding. As long as philosophy was confined to the definite attainment of the *know how* and the *do,* philosophy was an aid and not a hinderance, but when the course became inundated, as it were, with philosophy, the picture of the ideal was greatly obscured. If I may be pardoned, I believe I can make my meaning clearer by a personal illustration. My teacher of physiology was a most erudite man, so full of physiology and other sciences that to save his life he could not restrain himself to the bare, simple principles of physiology which my crude mind could hold and digest. From his stand-point he adequately covered the field and filled the measure of demand so full it overflowed at every side. He was absolutely drowned in the flood of physiology and all other *ologies* that inundated his mind. But one poor student found in a modest quiz-master, who only knew the headlines, the landmarks, the great and simple facts that beaconed the land of Physiology, his only salvation. The quiz-master knew only just enough to get to the end and go back again, refreshing the mind with the reiterated principles until all the salient facts of that most beautiful branch remain in my mind to this day like a twice-told tale. There you have it. Just so long as the teachers of dentistry had clear minds to teach the essential ideal of making a dental *practitioner,* just that long were they able to impart definite instruction which comparatively untrained minds could and did grasp and digest. In this connection, did it ever occur to you what the actual average of mental training among dental students is, or, more properly, was? The absolute, even sublime confidence with which the American dental student has always believed himself fitted to study any profession when he had got his own consent, is one of the enigmas of psychology. As I have already ruined my character, so to speak, I might as well utter another heresy, and that is, that it is a sacred tradition among the profession that every man is able and ought to criticise the dental colleges and be always willing to admit his own ability to flood with light all the dark problems that utterly confound and confuse them. Let me suggest that the man who belittles the colleges, as a rule, needs to take a course at one, or, at least, is ripe for a short post-graduate course. Can you make bricks without straw, or a silk purse out of a sow's ear? No; it is not that they do so little or so poorly, but the wonder is that they do so much with the material that you—the profession—send them.

To return to our mutton. I say it is no reasonable claim that the comparatively simple course of the earlier years was more appropriate to the simple and untrained minds which presented themselves at college, and, unless it can be clearly proved that the cultivation of the material now in the classes has proportionately improved, that the more complex courses, filled to bursting with excellent things, are still beyond the mental assimilation of the average student. There has been much improvement educationally, but not enough yet. The classes have grown younger and therefore more impressionable, which is a distinct gain, but receptivity of mind, engendered by training, must be attained before the best results can possibly be obtained.

Another point in our discussion. The mental attitude of the student has changed in late years. This is hard to prove, for so many personal exceptions suggest themselves that the mind cannot accept the principle broadly. In the earlier years, when there existed in the mind of the world the idea that dentistry was not quite as respectable as some other callings, the ranks of dentistry were recruited by those whose minds were more strongly directed than those of to-day. They answered, if you please, an outcry of their nature; they became dentists because they wanted to be dentists more than anything else in the world. To-day we have many who come into college because some single exponent known to them is supposed to be prospering greatly and yet can claim no superiority to them, who may be at the time "working themselves to death for nothing." This line of thought has been for quite a long time interesting to me, and if it were worth while many practically conclusive cases could be cited to prove its correctness. Now, everybody knows what the mental attitude means in the doing of anything. Did you ever hear of the forced march at night by the army of Northern Virginia, when the sticky clay fought with all its might to help the rebels hold back the advancing host? Did you ever hear that when mind as well as body was worn out, when sleep and rest seemed not only the greatest imaginable boon, but also the absolute necessity of the hour, that the band could change those dragging dead men into dancing figures, instinct with life and strength? That is a fact that has been often proved in the life of every man. The mental attitude of the self-elected, who listened to the outcry of nature, is infinitely superior to those, even better educated and more pliable of mind, who are actuated by a motive no higher than a commercial basis. If this is true, it can not be entirely unreasonable to claim that the average professional practitioner was better from that other *régime* than the average graduate of to-day who may be admitted to be a much fuller man. We cast no reflections upon the intrinsic worth of all scientific knowledge. We would not if we could say or do anything to minimize the just estimate placed upon the wonderful reaches of modern science. We would not have it thought that we would undervalue the great benefit that dentists may obtain from such broadest cultivation. The intelligent practitioner of dentistry cannot be too broad-minded nor too far-seeing to meet and solve the protean problems which daily confront him. The question is not how much has been supplied to him that was valuable, but rather how much knowledge has become the most wisdom to him? Knowledge is power, but it is only stored ammunition, passive potentiality; but wisdom is possibilities in action, mighty in effect for good or for evil. We must not measure nourishment by ingestion, but by digestion, and we would rest our claim, if we have any at all, for the correctness of my text upon the single idea that the average student in other days more nearly digested his course than is the case to-day. As far as this advocate is concerned, the proof is in and the jury has the matter for settlement. There is or is not some truth in the claim, and the prosecution has the opportunity to utterly annihilate the victim.

At the risk of outraging the unities, for fear of some mental reservations that may be formed or are in process of forming in your minds, I am going to say "what I have to say why sentence of death should not be pronounced upon me according to law." In the further language of Robert Emmett, "I have nothing to say that can alter your predetermination, or that would become me to say with any view to the mitigation of that sentence which you are here to pronounce and which I must abide by. But I have much to say why my reputation should be rescued from the load of calumny and false accusation that has been (in the last few minutes) heaped upon it." Therefore, and for that end, a few words will be pardoned. It will be recalled that I said that I had a growing conviction that the cart was before the horse in connection with our educational logic. It is but due to myself to make this a little clearer. As a practical teacher I have hailed each addition to the curriculum as another opportunity of value to the student. I have not always, however, felt certain that the practical value of the course would be necessarily increased by such valuable extension. The value is there,—that I did not question,—but I could not feel certain that its full cultural possibility would become tangible. Personally, I have been a stickler for broader, better education as the best foundation upon which to erect the superstructure of any professional equipment. Good education, however, is not measured by number, or length, or complexity of the courses, but by results, by increased power to think, by the cultivation of the powers of the mind, whether that has been accomplished by college methods and courses or not. The ability to grasp easily what is presented, to understand the teacher readily, not only as to what is said but what is meant, may be largely a matter of personality, but leading out the mental powers by education more certainly achieves that than anything else. Such skilled intelligence is what I have longed for and even hoped for in our classes. But now that condition of mental parts is most usually if not better obtained from collegiate instruction, and for that reason I have exulted when I saw that the trend of the action of the National Association of Dental Faculties was towards more definite educational instruction. Let me again use a homely figure. We may have the best seed as to quality, variety, and adaptability, and we may have in addition soil that promises the best results, but no farmer would hope to reap a harvest if he did not carefully prepare the ground. On the other hand, with thoroughly prepared earth fair results may be obtained even if it must be confessed that the farmer himself was not the wisest. The point I wish to make is that if we, the profession, can help the schools to so present the various subjects of the course, with especial relation to the preparation of the average student to understand the value of each part, this four-year law will certainly bring a great uplift to our professional future. Let us emphasize the importance of maturely weighing each branch and then intelligently placing it in the logical sequence of its relative importance to our scheme, and we shall greatly assist the cause of professional education. If we examine critically, we can show, I dare say, that most of our courses have become heterogeneous aggregations of material good enough in itself. The fault is rather in the lack of system, lack of logical sequence, rather than lack of satisfactory substance. Education-building is not altogether dissimilar to house-building so far as methodical sequence is concerned. It would be the grossest folly to attempt to put a part of the roof in the foundation, or, as I greatly fear we have been compelled to attempt to do, try to build the professional roof without any foundation under it. I would not have you suppose I undervalue the actual improvement of the course in the twenty-four years of my effort to teach, but in this heart-to-heart talk with you I want you to know that I have sometimes feared we were not getting the best results possible out of the opportunities we did have. This is said with special reference to those additions from the correlative sciences. With reference to those developments along the line of technic or the manual training in the art of dentistry, there can be no discussion. The inner consciousness of every single older dentist tells him that all of that most excellent practical cultivation would have been an inestimable boon to his young professional years. But I speak of those branches which from a broad scientific standpoint lend a distinct cultural insight into our special field of interest. If we could get all the maturing of mind and the training of intelligence desirable from their broadening influence, then all could build confidently; then we should reap the richest harvests or feel ourselves to blame for the deficiency.

This is the message I bring, a plea for a more logical rearrangement of the present curriculum, and thereby obtain a better use of all the excellent materials collated there, to the end that those entering our profession may, in fact as well as semblance, be *educated men.* Time is a very important factor in material digestion, and should not be deemed less so in the question of perfect educational assimilation. The failure to obtain satisfactory results is generally due to more than one cause. Starvation is most horrible when it is attended by the tantalizing association of copious ingestion of food, but it is possible. Therefore the addition of another session gives the assurance of a better opportunity than ever before to extract the full measure of professional nutrition from the courses in our colleges. Those with whom I am associated joined me in deeming the step to four sessions premature, but now that we have taken stock of our resources, we are determined to make the best effort possible to do better than ever. We are girding up our loins to meet the inevitable with the full consciousness that the schools stand committed to the new policy and should not back down without a manly effort to sustain its conclusion.

With the expression of my own personal opinion I beg to assure you that I am fully aware the last word has not been said. If, perchance, I have proved myself *flint* enough to strike the *spark* from you that shall light the torch of full, convincing, rational conclusion, I shall hold myself repaid better than I can adequately tell you. With this hope, I pray you to accept my thanks for your attention.

